# Recent Advances in Multi-Site Luminescent Materials: Design, Identification and Regulation

**DOI:** 10.3390/ma16062179

**Published:** 2023-03-08

**Authors:** Junhang Tian, Jihuan Xie, Weidong Zhuang

**Affiliations:** 1 State Key Laboratory of Advanced Metallurgy, University of Science and Technology Beijing, Beijing 100083, China; 2 School of Metallurgical and Ecological Engineering, University of Science and Technology Beijing, Beijing 100083, China; 3 Beijing Key Laboratory for Green Recovery and Extraction of Rare and Precious Metals, University of Science and Technology Beijing, Beijing 100083, China

**Keywords:** multi-site luminescence, emission regulation, rare earth luminescent materials, wLEDs

## Abstract

The development of novel phosphor materials with excellent performance and modification of their photoluminescence to meet the higher requirements for applications are the essential research subjects for luminescent materials. Multi-site luminescent materials with crystallographic sites for the activator ions that broaden the tunable range of luminescent spectra and even enhance the luminescent performance have attracted significant attention in the pursuit of high-quality luminescence for white light-emitting diodes. Here, we summarize multi-site luminescence characteristics based on the different kinds of host and activator ions, introduce the identifications of multi-site activator ions via optical analysis, provide a structural analysis and theoretical calculation methods, and introduce the regulation strategies and advance applications of multi-site phosphors. The review reveals the relationship between crystal structure and luminescent properties and discusses future opportunities for multi-site phosphors. This will provide guidance for the design and development of luminescent materials or other materials science.

## 1. Introduction

The Nobel Prize in Physics was awarded to Isamu Akasaki, Hiroshi Amano and Shuji Nakamura “for the invention of efficient blue light-emitting diodes (LEDs) which has enabled bright and energy-saving white light sources” in 2014. The white LEDs (wLEDs) have been widely used as the fourth generation of green solid-state lighting sources in general lighting, displays and other advanced applications [1,2]. To achieve high-quality white lighting, phosphor-converted wLEDs (pc-wLEDs) with high luminescence efficiency, stable color, spectral design flexibility and low cost have become the mainstream [3,4]. Nowadays, the luminous efficacy of commercial pc-wLEDs has been improved repeatedly. As one of the core luminescent materials of pc-wLEDs, phosphors determine the color quality and application performance of the solid-state lighting devices. An excellent phosphor with strong absorption, large excitation band, tunable emission band, high luminous efficiency, low thermal quenching, high chemical and thermal stability, appropriate particle morphology, etc. is required [5]. Hence, the design and development of high-quality phosphors are an eternal subject in the field of solid-state lighting.

To meet higher requirement of pc-wLED applications, more multi-site luminescent materials have been focused on and investigated by increasing numbers of scientists and researchers. Previously, the widely used pc-wLEDs combined with a blue chip and yellow emitting Y_3_Al_5_O_12_:Ce^3+^ were restricted to low color rendering index (CRI) Ra < 80 for advanced and healthy applications [6]. As such, high-quality full-spectrum wLEDs that simulated the solar spectrum have been proposed, leading to a variety of luminescent materials with broadband emission and excellent luminescent properties being developed. Among them, the various multi-site phosphors with blue, cyan, green, yellow, and red emissions exhibit a broadened and flexibly tunable spectrum and have been widely used to obtain excellent CRI. Also, the multi-site phosphors can reduce the variety of luminescent materials, thereby decreasing the re-absorption phenomenon, fabrication complexity and color drift due to different thermal behavior between various phosphors, ensuring that the assembled wLEDs show the desired luminescence performance [7]. Moreover, with the rapid demand of near-infrared (NIR) lighting, the multi-site NIR phosphors with broadband emission have attracted significant attention due to various important applications requiring different wavelength ranges [8]. The Cr^3+^, Mn^2+^ and Eu^2+^ activator ions are introduced into multiple crystallographic sites to construct significant multi-site NIR phosphors for practical applications. No matter what the light color of the phosphors, their emission bands are closely related to the lattice environments, where activator ions dominated, as well as their nearest neighbor sites. The excited electrons of activator ions transit to their ground states and release energy in the form of emission or lattice vibrations [9,10]. As such, the design and regulation of luminescent properties are directly dependent on the effect of lattice vibrations and localized crystallographic structure. According to the urgent requirements for better performance, the band gap engineering and crystal-site/phase engineering strategies are equipped to regulate and modify the multi-site phosphor for the benefit of human life.

Over the past few years, lots of investigations based on multi-site luminescence have been reported. However, scarcely any reviews focusing on the subject have been published. Our review will summarize the relevant research concerning the development and modification of multi-site luminescent materials. Based on the different kinds of host and activator ions, the multi-site luminescence characteristics and their relationship between crystal structure and luminescence will be introduced in detail. Identification of the preferential occupation for the activator ions in multi-site phosphors will be evaluated and elucidated in terms of optical analysis, structural analysis and theoretical calculations. The modification of the emission spectrum, enhancement of luminescence performance and advanced applications for multi-site phosphors will be described clearly. These summaries and comments will try to analyze the influence mechanism of multi-site-doping activator ions on the structure and luminescence properties, reveal the relationship between crystal structure and luminescent properties and provide guidance for the design and modification of multi-site phosphors.

## 2. Multi-Site Luminescence of Phosphors

The classic phosphor system is composed of the host and the activator ions [9]. The host material constructs the bulk of phosphor, that provides proper crystallographic sites for activator ions. The activator ions, which are doped into the host lattice in relatively small amounts, can absorb excitation energy and convert it into the ultraviolet, visible or infrared emission. In general, the host is optically inert, but its local structure affects the electronic structure of the activator ions so that different activators dominated at different crystallographic sites exhibit varying multi-site luminescence. To sufficiently describe the multi-site luminescence of phosphors, the construction of multisites in the host and the luminescent properties of different activator ions have been discussed and are clearly explained in this section.

### 2.1. Construction of Multisites in Host

**Figure 1:**
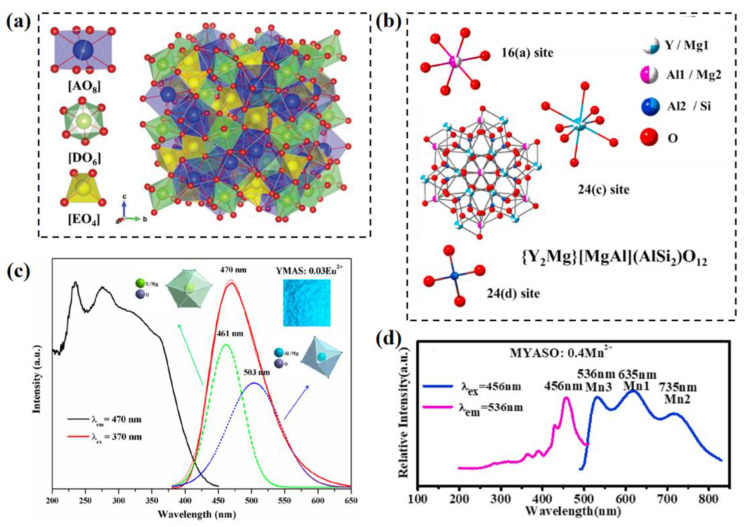
(**a**) The general structure model of garnet. Reprinted with permission from ref. [13]. Copyright 2021 Royal Society of Chemistry. (**b**) Crystal structure model of {Y_2_Mg}[MgAl](AlSi_2_)O_12_. Reprinted with permission from ref. [14]. Copyright 2021 Elsevier B.V. (**c**) Excitation (λ_em_ = 470 nm) and emission (λ_ex_ = 370 nm) spectra of YMAS:0.03Eu^2+^ phosphor. Reprinted with permission from ref. [15]. Copyright 2019 American Chemical Society. (**d**) Excitation and emission spectra of YMAS:0.4Mn^2+^ phosphor. Reprinted with permission from ref. [14]. Copyright 2021 Elsevier B.V (Amsterdam, The Netherlands).

The design of phosphors based on the typical structural model is an effective strategy [11]. To achieve multi-site luminescence, the host of the phosphor should provide various crystallographic sites for activator ions. At present, many kinds of compounds that accommodate various cations with different valence states have been designed and developed for a variety of phosphors with multi-site luminescence, including garnet-structure, apatite-structure, melilite-structure, A_3_MX_5_-structure, β-K_2_SO_4_-structure, whitlockite (β-Ca_3_(PO_4_)_2_)-structure phosphors, etc. Among them, garnet-structure phosphors have been widely used in wLEDs due to their excellent structural flexibility, good physical and chemical stability and high luminescent efficiency. The classic Y_3_Al_5_O_12_:Ce^3+^ (YAG:Ce^3+^) phosphor was synthesized over fifty years ago [12]. Garnet-structure compounds have the general formula of A_3_D_2_E_3_O_12_; their structure model and the [AO_8_] dodecahedrons, [DO_6_] octahedrons, and [EO_4_] tetrahedrons are illustrated in Figure 1a [13]. As with most of garnet phosphors that have been reported, Ce^3+^ is generally dominated at a single crystallographic site, and even Eu^2+^ is difficult to introduce into the garnet lattice; this can be attributed to the significant differences in the crystallographic environment among the A, D and E sites and the rare appropriate divalent crystal sites in the garnet lattice. As a result of the research and development of the garnet-structure phosphor, increasing numbers of multi-composition materials are synthesized and studied, as the A, D and E cationic sites can be occupied by various divalent, trivalent or tetravalent ions, including Y^3+^, La^3+^, Gd^3+^, Lu^3+^, Mg^2+^, Ca^2+^, Sr^2+^, Ba^2+^, Al^3+^, Ga^3+^, Sc^3+^, Zr^4+^, Hf^4+^, Si^4+^, Ge^4+^, etc. The garnet lattice possesses various cationic crystallographic sites with different valence states, which provide significant possibilities to construct numerous multi-site luminescence opportunities. 

To introduce divalent site and construct multisites for Eu^2+^ doping, the {Y_2_Mg}[MgAl](AlSi_2_)O_12_ (YMAS) is designed via the substitution of Mg^2+^-Si^4+^ for Y^3+^-Al^3+^ and Al^3+^-Al^3+^ in YAG, whose structure model is illustrated in Figure 1b [14]. The emission spectrum, excited at 370 nm of YMAS:Eu^2+^, exhibits a broad band that can be divided into two Gaussian bands peaked at 461 nm and 503 nm due to the Eu^2+^ ions dominated at eight-coordinated and six-coordinated crystallographic sites, respectively (as shown in Figure 1c) [15]. The introduction of Mg^2+^ into the A and D sites of the garnet lattice provides suitable crystallographic multisites for the Eu^2+^ activator ion, resulting in the sensitive regulation of color point via tuning the doping concentration of Eu^2+^. Furthermore, the full width at half maximum (FWHM) is changed from 80 to 105 nm with the Eu^2+^ doping level from 0.01 to 0.10 due to the crystal field splitting and nephelauxetic effect. Similarly, the Mn^2+^ doped YMAS phosphor is synthesized, which exhibits the multiple emissions. As shown in Figure 1d, the Mn^2+^ ions occupy Mg^2+^ site of [AO_8_] dodecahedron to form red emission peaked at 635 nm (Mn1), replace Mg^2+^ site of [DO_6_] octahedron to form deep red emission peaked at 735 nm (Mn2) and dominate the Al^3+^ site of [EO_4_] tetrahedron to form green emission peaked at 536 nm (Mn3) [14]. The YMAS:Mn^2+^ phosphor shows broadband emission covering green-red-deep red regions; this is an excellent candidate for full-spectrum plant growth LEDs. Recently, many novel garnet-structure phosphors with multi-site luminescence have been developed via activator ions doping and co-doping strategy. The activator ions include, but are not limited to, Ce^3+^, Eu^2+^, Bi^3+^, Eu^3+^, Mn^2+^, Dy^3+^, etc. [16,17,18,19,20,21] Various luminescence and its modification are achieved due to the multi-site doping, local structure tuning and energy transfer.

**Figure 2:**
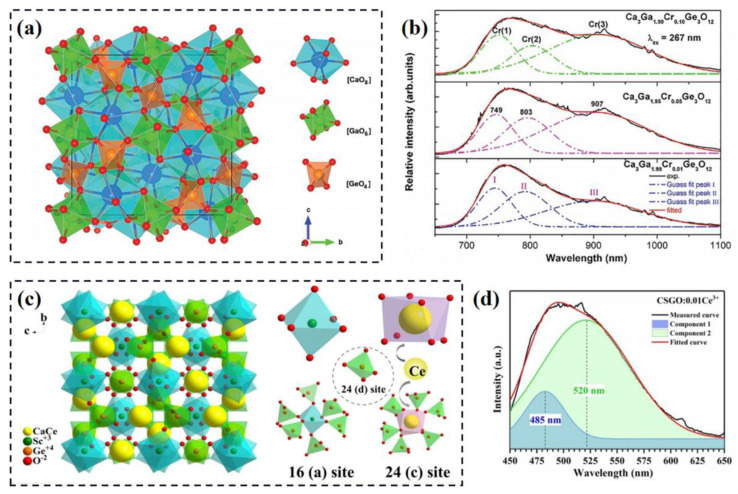
(**a**) Schematic diagram of Ca_3_Ga_2_Ge_3_O_12_ structure and coordination environment of Ca^2+^, Ga^3+^ and Ge^4+^ cations. (**b**) The NIR emission spectra of the samples Ca_3_Ga_2−x_Ge_3_O_12_ (x = 0.01, 0.05, 0.10, respectively) under 267 nm excitation at room temperature. Reprinted with permission from ref. [22]. Copyright 2017 WILEY-VCH Verlag GmbH and Co. KGaA, Weiheim. (**c**) Crystal structure of Ca_3_Sc_2_Ge_3_O_12_:Ce^3+^ phosphor. (**d**) The decomposed photoluminescence spectrum of Ca_3_Sc_2_Ge_3_O_12_:0.01Ce^3+^ phosphor by Gaussian fitting method. Reprinted with permission from ref. [23]. Copyright 2019 Royal Society of Chemistry (London, UK).

In addition to yttrium aluminum garnet phosphor, the Si-series and Ge-series garnet phosphors have attracted significant attention as luminescent materials for wLEDs, including Ca_3_Sc_2_Si_3_O_12_:Ce^3+^, Mg_3_Y_2_Ge_3_O_12_:Ce^3+^, etc. When Ca^2+^ dominates the A site, Ga^3+^ dominates the D site and Ge^4+^ dominates the E site, the Cr^3+^-doped Ca_3_Ga_2_Ge_3_O_12_ garnet-structure phosphor with super broadband near-infrared (NIR) emission is obtained (structure model of host and emission spectra are shown in Figure 2a,b [22]). The emission spectra can be deconvoluted into three Gaussian fitted peaks, dominated around 749 nm (Cr1), 803 nm (Cr2) and 907 nm (Cr3), respectively. The Cr1 emission corresponds to the Cr^3+^ at the Ga^3+^ site, and the Cr2 and Cr3 emissions are attributed to Cr^3+^(Ca^2+^)-Ga^3+^(Ge^4+^) associations as defect sites. Similarly, the vibration-coupling structure is observed in the Ca_3_Sc_2_Ge_3_O_12_:Ce^3+^ (CSGO:Ce^3+^) phosphor (structural model and emission spectrum are illustrated in Figure 2c,d [23]). Although the activator ion is dominated at only one crystallographic site, two luminescence centers of Ce^3+^ are observed, peaked at 490 nm (Ce1) and 530 nm (Ce2), respectively. The Ce1 emission is ascribed to the intrinsic luminescence of Ce^3+^ in dodecahedron, while the Ce2 emission that does not depend on doping concentration is attributed to the formation of Ce^3+^ pairs. Energy transfer from Ce1 to Ce2 and good thermal stability are observed for CSGO:Ce^3+^. Hence, the formation of dissimilar local structure is an effective strategy to construct multi-sites for activator ions in the host lattice. According to this method, a series of garnet-structure phosphors with varying local structure and at least two luminescent centers are designed and synthesized, such as Ce^3+^-doped Y_3_MgAlAl_2_SiO_12_, CaY_2_Al_4_SiO_12_, etc. [24,25]. The enhancement of emission intensity and thermal stability, as well as the peak shift and a considerable broadening of the emission band, can be achieved.

**Figure 3:**
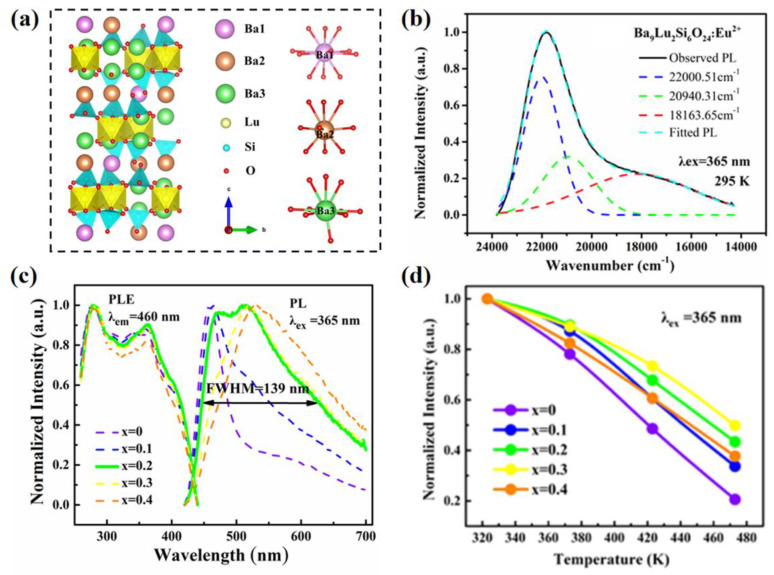
(**a**) Crystal structure of Ba_9_Lu_2_Si_6_O_24_ and the coordinated environment of Ba1, Ba2, Ba3 and Lu. (**b**) Normalized emission spectrum and Gaussian fitting spectra excited at 365 nm of Ba_9_Lu_2_Si_6_O_24_:Eu^2+^ sample at 295 K. (**c**) Normalized emission spectra (λ_ex_ = 365 nm), excitation spectra (λ_em_ = 460 nm) and (**d**) temperature-dependent intensity of emission spectra of (Ba_1−x_Sr_x_)_9_Lu_2_Si_6_O_24_:Eu^2+^ (x = 0, 0.1, 0.2, 0.3 and 0.4) samples at 295 K. Reprinted with permission from ref. [26]. Copyright 2022 American Chemical Society (New York, NY, USA).

Just like garnet-structure phosphors, as well as the compounds that possess various cations with various valence states, the host can be occupied by the activator ions for various cationic elements. Furthermore, some lattices only obtain single cationic element that is suitable for the activator ions, but the single cationic element dominates various crystallographic sites, which can also result in multi-site luminescence. As for the Ba_9_Lu_2_Si_6_O_24_:Eu^2+^ phosphor (structural model of host is illustrated in Figure 3a), the Eu^2+^ activator ion only can be substituted with Ba^2+^ ions due to the ionic radii and valence state in the lattice [26]. However, the Ba^2+^ ions dominate three independent crystallographic sites, so that the phosphor exhibits asymmetrical broadband emission, as shown in Figure 3b, which can be divided into three emission bands peaked at 455, 478 and 551 nm due to the Eu^2+^ at Ba1, Ba2 and Ba3 sites, respectively. With Sr-substitution in the lattice, the preferential occupation of Eu^2+^ is changed between the three cationic crystallographic sites, which results in the regulation of the emission band, as shown in Figure 3c. The (Ba_0.8_Sr_0.2_)_9_Lu_2_Si_6_O_24_:Eu^2+^ phosphor shows a super broad band with FWHM of 139 nm. The Sr-substituted samples show better emission intensity and thermal stability. The temperature-dependent intensities of the samples are shown in Figure 3d, and are discussed in detail in Section 4.2. Hence, selecting a host lattice with various suitable cationic crystallographic sites for activator ions, whether these multi-sites are provided by multiple or single elements, is the essential factor to achieve multi-site luminescence. Through the composition tailoring, energy transfer and other modification strategies, the design of novel phosphors, regulation of emission and enhancement of luminescent properties can be achieved.

### 2.2. Luminescent Properties of Different Activator Ions

Different activator ions doped into a multi-site host lattice will exhibit different luminescence. This section introduces various multi-site luminescence based on different activator ions, including Eu^2+^, Ce^3+^, Cr^3+^, Eu^3+^, Mn^2+^, Mn^4+^, Bi^3+^, etc.

#### 2.2.1. Eu^2+^

**Figure 4:**
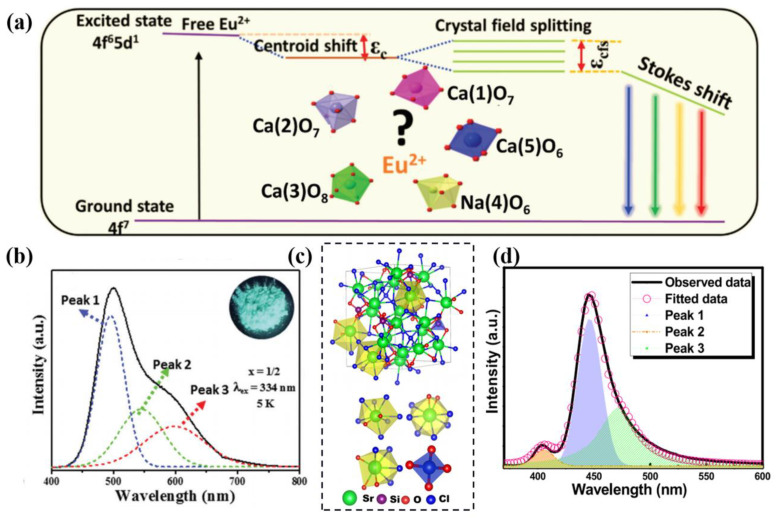
(**a**) Schematic diagram illustrating the influence of the crystal environment on the 5d energy levels of Eu^2+^ doped in β-Ca_3_(PO_4_)_2_-type compounds. (**b**) Gaussian fitting results of PL spectra for Ca_9_Na_3/2_Y_1/2_(PO_4_)_7_:Eu^2+^. Reprinted with permission from ref. [41]. Copyright 2021 Royal Society of Chemistry. (**c**) Illustration of the crystal structure and Sr-polyhedral units of Sr_5_SiO_4_Cl_6_. (**d**) Room-temperature PL emission spectrum and three Gaussian curves of Sr_4.9_Eu_0.1_SiO_4_Cl_6_. Reprinted with permission from ref. [46]. Copyright 2019 American Chemical Society.

The divalent Eu ion is a common activator for rare earth luminescent materials, whose 4f-5d transition is illustrated in Figure 4a. The electrons of Eu^2+^ in the 4f^7^ ground state are excited to the 4f^6^5d^1^ excited state to form the free Eu^2+^ ion. The centroid shift ε_c_ is related to the cation electronegativity and polarizability of anionic ligands. The crystal field splitting ε_cfs_ is inversely proportional to the distance between the center ion and the ligand. Hence, the excitation and emission of Eu^2+^ is directly affected by the crystal environment where it dominates [27]. Some Eu^2+^-doped multi-site phosphors are summarized for the host, multiple crystallographic sites for Eu^2+^ and its corresponding emission peaks and FWHM, as shown in Table 1.

**Table 1:** Summary of structural and luminescent information for some Eu^2+^-doped multi-site phosphors.

Host	Multisites (The Emission Peaks)	FWHM	Reference
{Y_2_Mg}[MgAl](AlSi_2_)O_12_	Y/Mg (461 nm), Al/Mg (503 nm)	80–105 nm	[14]
(Sr,Ba)_2_SiO_4_	Sr1 (450 nm), Sr2(490 nm)	-	[28]
Ba_1+y_Sr_1-y_Ga_4_O_8_	Ba/Sr1, Ba/Sr2, Ba/Sr3	140–230 nm	[29]
Sr_3_SiO_5_	two sites (485, 579 nm)	-	[30]
Sr_3_Si_2_O_4_N_2_	Sr4, Sr5, Sr6 (600 nm)	≈80 nm	[31]
Sr_3_Si_5.5_Al_2.5_O_9.5_N_5.5_	Sr1 (455 nm), Sr2 (493 nm)	70 nm	[32]
Ca_3_MgSi_2_O_8_	Ca1 (476 nm), Ca3 (503 nm)	2967 cm^−1^	[33]
Ba_9_Lu_2_Si_6_O_24_	Ba1 (457 nm), Ba2 (478 nm), Ba3 (624 nm)	139 nm (Sr-doped-0.2)	[26]
Ba_2_CaB_2_Si_4_O_14_	Ba (408 nm), Ca (548 nm)	-	[34]
Li_4_SrCa(SiO_4_)_2_	Sr (425 nm), Ca (575 nm)	-	[35]
Ca_6_BaP_4_O_17_	Ba (390 nm), Ca1 (523 nm), Ca2 (569 nm)	-	[36]
RbBaPO_4_	Ba (406 nm), Rb (431 nm)	-	[37]
BaSrMg(PO_4_)_2_	Ba^2+^(BaO7), Sr^2+^(SrO7), Ba^2+^(BaO8), Sr^2+^(SrO8)	163 nm	[38]
KBa_2_(PO_3_)_5_	K1 (430 nm), Ba1 (480 nm), Ba2(542 nm)	160 nm	[39]
Ca_10_Na(PO_4_)_7_	Ca1/Ca3 (407 nm), Ca2 (483 nm), Na (573 nm)	-	[40]
Ca_9_Na_3/2_Y_1/2_(PO_4_)_7_	Ca3 (495 nm), Ca1/Ca2 (550 nm), Na4 (600 nm)	-	[41]
Sr_8_CaAl(PO_4_)_7_	Sr1/Sr3/Sr5 (507 nm), Sr2 (555 nm), Sr4 (610 nm)	-	[42]
(SrCa)_2_La(PO_4_)_3_O	Sr/Ca/La1 (452–463 nm), Sr/Ca/La2 (497–517 nm)	72 nm	[43]
K_3_La(Ca)(PO_4_)_2_	K1 (570 nm), K2 (517 nm), K3 (430 nm), La (620 nm)	-	[44]
LiSr_4_(BO_3_)_3_	Sr1 (625 nm), Sr2 (711 nm)	123 nm	[45]

Generally, Eu^2+^ prefers to occupy the divalent alkaline earth metal cation sites due to the appropriate valence state and ionic radius. As for the β-Ca_3_(PO_4_)_2_-structure compounds, when the Y^3+^-Na^+^ substitutes the Ca^2+^-Ca^2+^, the Eu^2+^ ions could possibly dominate five kinds of polyhedrons, such as Ca(1)O_7_, Ca(2)O_7_, Ca(3)O_8_, Ca(5)O_6_ and/or Na(4)O_6_ [41]. The emission spectrum of the Ca_9_Na_3/2_Y_1/2_(PO_4_)_7_:Eu^2+^ phosphor exhibits an asymmetric peak that can be divided into three Gaussian fitting bands, as shown in Figure 4b. According to the calculation of 4f-5d transition of each site based on the empirical formula for the emission and absorption energy, the three sub-bands are attributed to Eu^2+^ ions occupied at the Ca(3) site (peaked at 495 nm), the Ca(1) and (2) sites (peaked at 550 nm) and the Na(4) site (peaked at 600 nm). The Ca(1) and Ca(2) sites have the same valence bond sum and coordination environment, which results in similar centroid shift (ε_c_) and crystal field splitting (ε_cfs_) and emission bands for Eu^2+^ at these two sites; as such, these sites can be considered as the same position. With the introduction of Y^3+^-Na^+^ into the lattice, another suitable crystal environment is created for Eu^2+^-doping, meaning that non-equivalent substitution is feasible for the construction of multi-site luminescence.

Activator ions doping into multisites commonly result in a broadband emission due to the recombination of several sub-bands of the multiple luminescence. The Eu^2+^ typically exhibits broadband emission due to its 4f-5d allowed transition characteristics. Only a few narrow-band Eu^2+^-activated phosphors have been developed, including β-sialon:Eu^2+^. Based on the above opinions, the narrow-band multi-site phosphor seems impossible. However, if Eu^2+^ has a significantly different occupation preference for different crystallographic sites, and the preferential occupation has high lattice rigidity and symmetry, the multi-site narrow-band Eu^2+^-activated phosphors can be obtained. For Eu^2+^-activated Sr_5_SiO_4_Cl_6_, the host provides three kinds of Sr polyhedrons for the activator, and their occupation preference is Sr(3)O_8_ > Sr(1)O_9_ > Sr(2)O_7_ (structural model is illustrated in Figure 4c [46]). The emission bands are deconvoluted to three sub-bands peaked at 472 nm (Sr1 site), 445 nm (Sr3 site) and 406 nm (Sr1 site), as shown in Figure 4d. The highly symmetric crystal environment of Eu^2+^ results in an FWHM of 33 nm, color purity above 90%, internal quantum efficiency of 91.4% and an excellent thermal stability.

#### 2.2.2. Ce^3+^

**Figure 5:**
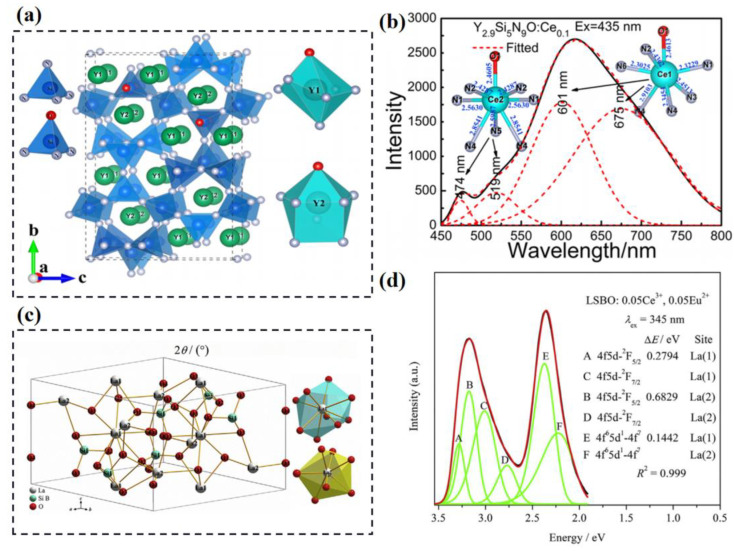
(**a**) Crystal structure of Y_3_Si_5_N_9_O and coordination polyhedral of Y1 and Y2. (**b**) PL emission spectrum of Y_2.9_Si_5_N_9_O:Ce_0.1_ under the excitation of 435 nm radiation. Reprinted with permission from ref. [47]. Copyright 2016 American Chemical Society. (**c**) Approximate crystal structure of La_5_Si_2_BO_13_. (**d**) The detailed Gaussian fitting results of La_5_Si_2_BO_13_:0.05Ce^3+^, 0.05Eu^2+^ sample. Reprinted with permission from ref. [48]. Copyright 2021 Elsevier B.V.

In a similar fashion to Eu^2+^, the luminescence of Ce^3+^ shows 4f-5d allowed transition characteristics. However, two types of 4f ground energy levels (^2^F_7/2_, ^2^F_5/2_) lead to the asymmetric emission band of Ce^3+^, even if the activator ions only dominate a single crystallographic site; as such, the multi-site luminescence of Ce^3+^ exhibits more abundant optical performance. Figure 5a shows the structural model of an orthorhombic-structure oxynitride Y_3_Si_5_N_9_O with two kinds of crystallographic sites for Y^3+^; this creates two different distorted polyhedrons: Y1(N,O)_7_ and Y2(N,O)_8_ [47]. As shown in Figure 5b, the emission spectrum of Y_3_Si_5_N_9_O:Ce^3+^ can be divided into four Gaussian sub-bands peaked at 474, 519, 601 and 675 nm, respectively. The former two sub-bands are attributed to Ce^3+^ dominated at the Y2 site, while the latter two sub-bands are ascribed to Ce^3+^ dominated at the Y1 site. The Ce^3+^-doped Y_3_Si_5_N_9_O phosphor shows an extreme broadband emission, covering from blue to deep red, with a large FWHM of 178 nm. The external and internal quantum efficiencies and absorption are 15.6, 17.2 and 89.5% under 450 nm excitation, respectively. Some Ce^3+^-doped multi-site phosphors are summarized and shown in Table 2.

Co-doping several activator ions in a multi-site host is an effective strategy to construct various luminescent centers and energy transfer, which can realize the enhancement of luminescent performance. Eu^2+^ and/or Ce^3+^ are commonly co-doped together or with other activator ions to develop novel optical materials. The La_5_Si_2_BO_13_ lattice with an apatite structure provides two kinds of La sites for the Eu^2+^ and Ce^3+^ activator ions, as illustrated in Figure 5c [48]. Under the excitation of 345 nm, the emission spectrum of La_5_Si_2_BO_13_:Ce^3+^, Eu^2+^ phosphor exhibits a bimodal asymmetric band that can be deconvoluted into six Gaussian fitted peaks, as shown Figure 5d. The A, B, C and D sub-bands correspond to the transitions of Ce^3+^ (5d→^2^F_7/2_, ^2^F_5/2_) dominated at different two La crystallographic sites, as the E and F sub-bands belong to the emissions of Eu^2+^ at the La1 and La2 sites, respectively. The energy transfer from Ce^3+^ to Eu^2+^ is observed in the co-doped phosphor. The Ce^3+^ and Eu^2+^ ions show diverse responses with temperature change, making the La_5_Si_2_BO_13_:Ce^3+^, Eu^2+^ phosphor a potential candidate for optical thermometry. The relevant application of multi-site luminescence in relation to temperature or even pressure sensing will be introduced in next section. In addition, some other co-doped multi-site luminescent materials have been designed and reported that are not described further. These materials have co-doped activator ions that include, but not limited to, Ce^3+^, Eu^2+^, Tb^3+^, Mn^4+^, Eu^3+^, Sm^3+^, Bi^3+^, etc. [54,55,56,57,58].

#### 2.2.3. Cr^3+^

Nowadays, NIR light sources have been attracted significant attention in applications ranging from security monitoring, food testing, biological recognition, vivo imaging, light touch switches, smoke alarms and others [59]. NIR phosphor-converted LEDs have been widely adopted due to their broadband spectra, better wavelength-tunable performance and low cost. Consequently, the design and development of high-quality NIR phosphors has become an essential research subject. Because of the suitable transition characteristics of Cr^3+^, a variety of Cr^3+^-doped phosphors have been studied and used to produce NIR light over the past few years; these include Gd_3_Sc_1.5_Al_0.5_Ga_3_O_12_:Cr^3+^ [60], Na_3_ScF_6_:Cr^3+^ [61] and ScBO_3_:Cr^3+^ [62], among others. However, their PL wavelength needs to be longer, and their FWHM needs to be wider, in order to be more suitable for the various applications mentioned above. Therefore, constructing multi-site luminescence and energy transfer are the effective strategies for enhancing the luminescence properties of Cr^3+^-activated phosphors for NIR light.

**Figure 6:**
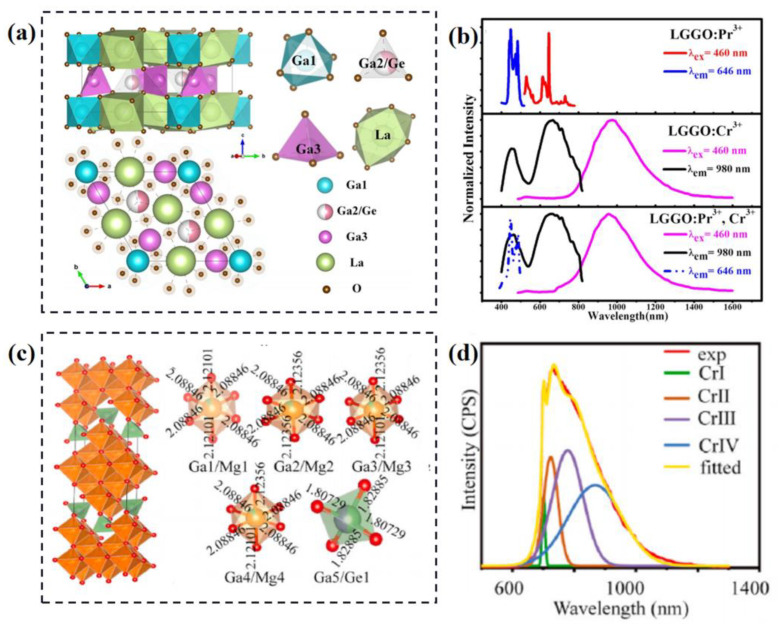
(**a**) Unit cell structure of LGGO and coordination environment of cations. (**b**) PL and PLE spectra of LGGO:Pr^3+^, LGGO:Cr^3+^ and LGGO:Pr^3+^, Cr^3+^. Reprinted with permission from ref. [63]. Copyright 2019 American Ceramic Society. (**c**) Crystal structure of Mg_7_Ga_2_GeO_12_ and coordination environment of Mg, Ga and Ge. (**d**) Gaussian fitting for the PL spectrum of Mg_7_Ga_2_GeO_12_:0.02Cr^3+^ into four peaks. Reprinted with permission from ref. [64]. Copyright 2021 Elsevier Ltd. and Techna Group S.r.l.

The gallate La_3_Ga_5_GeO_14_ (LGGO), with multiple cationic crystallographic sites, is an appropriate host for Cr^3+^-doped luminescent materials; it has been widely studied over the years. Figure 6a shows the structural model of LGGO host; it includes three different Ga sites with different coordination numbers and the crystal environment of La and Ge [63]. Considering the differences of valance states and ionic radii between Cr^3+^ and La^3+^/Ga^3+^/Ge^4+^, the activator ions are prone to substitute Ga^3+^ ions. The preferential occupation of Cr^3+^ is identified based on the EPR measurement, Rietveld refinements of XRD and First-principles DFT calculations. The results show that the activator ions prefer to dominate at the Ga1 and Ga3 sites. The identification methods of multisites of the activator ions will be described in detail in Section 3. The multi-site luminescence for LGGO:Cr^3+^ phosphor is traced; it shows a super broadband NIR emission peaked at 980 nm with a large FWHM of 330 nm, as shown in Figure 6b. To optimize the application performance, the energy transfer is constructed via Pr^3+^-Cr^3+^ co-doping, as the Pr^3+^ acts as a sensitizer, so that the luminescence intensity of co-doped sample is improved 3 times. The internal quantum yield of the Cr^3+^-activated sample is 7.7%, and that of the co-doped sample is 38.5%. La_3_Ga_5_GeO_14_:Pr^3+^, Cr^3+^ exhibits significant potential as a broadband NIR phosphor for application in food testing.

Recently, many Cr^3+^-activated phosphors with ultra-broadband emission resulting from multi-site luminescence have been developed. The Cr^3+^ can be dominated at five different kinds of crystallographic sites of Mg_7_Ga_2_GeO_12_, as illustrated in Figure 6c [64]. The emission spectrum of Cr^3+^-doped Mg_7_Ga_2_GeO_12_ can be deconvoluted into four individual Gaussian bands peaked at 700 nm (CrI), 723 nm (CrII), 778 nm (CrIII) and 867 nm (CrIV), which are attributed to the activator dominated at [Ga1/Mg1], [Ga3/Mg3, Ga4/Mg4], [Ga2/Mg2] and [Ga5/Ge1] sites, respectively. Furthermore, the concentration quenching of different emissions shows different speeds (CrI > CrII > CrIII > CrIV) which also causes energy transfer. Based on the above, the FWHM of Mg_7_Ga_2_GeO_12_:Cr^3+^ phosphor can be broadened from 101 to 226 nm by changing the Cr^3+^ doping concentration. Similarly, some Cr^3+^-doped multi-site NIR phosphors are summarized and shown in Table 3.

#### 2.2.4. Eu^3+^

Due to its f-f forbidden transition characteristics, the Eu^3+^ emission exhibits a series of sharp emission bands whose peaks barely shift with changes in the local surrounding. However, the multi-site regulation of Eu^3+^ can achieve the modification of color coordinates, luminescence efficiency and thermal stability. For instance, the SrLu_2_O_4_ (SLO) compound with an orthorhombic structure provides two inequivalent crystallographic sites of Lu^3+^ that can be dominated by Eu^3+^ activator ions, forming the two types of (Lu1/Eu1)O_6_ and (Lu2/Eu2)O_6_ octahedrons [76]. The SLO:9%Eu^3+^ phosphor shows red emission with five primary sharp peaks at 581, 593, 611, 653 and 708 nm, corresponding to the ^5^D_0_→^7^F_J_ (J = 0, 1, 2, 3, 4) transitions, respectively. When the Eu^3+^ doping concentration increases, the luminescent performance is integrally enhanced due to the preferential occupation and energy transfer between the Eu^3+^ ions at multisites in the lattice. As the Eu^3+^ doping concentration is 9%, the phosphor shows the maximum emission intensity, the closest color coordinates with the standard red light as (0.646, 0.354) and excellent thermal stability as the emission intensity at 423 K remains 89% of that measured at room temperature. Various crystallographic sites for Eu^3+^ provide suitable modification and optimization of the multi-site luminescence to realize high-quality applications of the novel phosphors [77,78].

**Figure 7:**
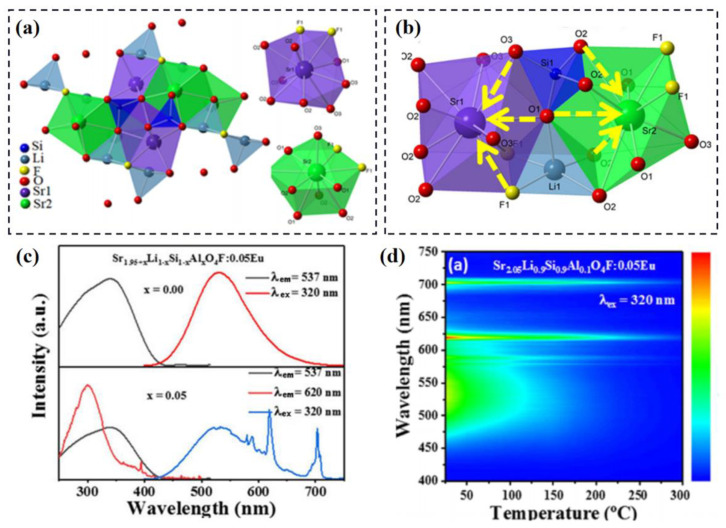
(**a**) Crystal structure of Sr_2_LiSiO_4_F. (**b**) Schematic diagram of the local structure change of Sr site. (**c**) PLE and PL spectra of Sr_1.95+x_Li_1−x_Si_1−x_Al_x_O_4_F:0.05Eu (x = 0 and 0.05). (**d**) Temperature-dependent PL spectra of Sr_1.95+x_Li_1−x_Si_1−x_Al_x_O_4_F:0.05Eu (x = 0.10). Reprinted with permission from ref. [79]. Copyright 2022 American Chemical Society.

Furthermore, Eu is a lanthanide element with two different valence states, that exhibits various types of emission in the luminescent materials. The valence-state transfer between divalent and trivalent Eu ions in a single-phase multi-site phosphor is a promising strategy to obtain warm white light. The luminescence can be modified via the structural evolution of the multi-site compounds. Figure 7a illustrates the structure model of Sr_2_LiSiO_4_F with two kinds of Sr sites [79]. Due to the substitution of the Sr^2+^-Al^3+^ pair by the Li^+^-Si^4+^ pair, the local structure of Sr sites where activator ions are doped is changed, as shown in Figure 7b. This results in the compression of Eu^2+^-occupation and the stabilization of Eu^3+^-occupation at the Sr sites, especially in the reducing atmosphere. As for the luminescence performance shown in Figure 7c, the Sr_2_LiSiO_4_F:Eu phosphor exhibits broadband green emission due to the characteristic transition of Eu^2+^. After the cation-pair substitution, the Sr_1.95+x_Li_1-x_Si_1-x_Al_x_O_4_F:0.05Eu (x = 0.05) phosphor shows not only broadband green emission that is similar to the x = 0 sample, but also a series of sharp-line emissions due to the characteristic transition of Eu^3+^. The coexistence of Eu^2+^ and Eu^3+^ and the energy transfer between them result in the various emission caused by different activated wavelengths. This shows their potential for anti-counterfeiting applications. Also, the diverse thermal response emissions of mixed-valence Eu ions, as illustrated in Figure 7d, make it possible to use the co-doping sample for optical thermometry. Hence, constructing multi-site luminescence of Eu with multiple valence states is a feasible method to design a novel phosphor for advanced applications [57,80]. Eu^3+^ can be applied as the structural probe to identify multi-site luminescence for other kinds of activator ions; this will be introduced in next section [81].

#### 2.2.5. Mn^2+^, Mn^4+^

**Figure 8:**
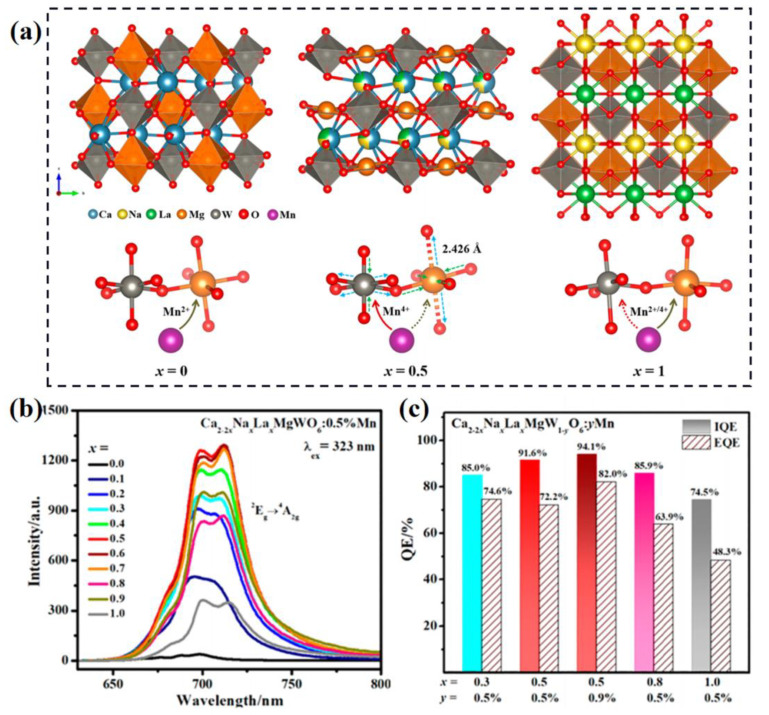
(**a**) Crystal structure diagram of Ca_2−2x_Na_x_La_x_MgWO_6_ (x = 0, 0.5, 1.0), and the schematic diagram of site occupancy preference of Mn and the resulting +2/+4 valence stability. (**b**) The PL spectra and (**c**) QE values of Ca_2−2x_Na_x_La_x_MgWO_6_:Mn samples. Reprinted with permission from ref. [84]. Copyright 2022 American Chemical Society.

As with Eu ions, Mn ions with two types of valance state can be used as the activator of phosphors, including Mn^2+^ and Mn^4+^. When doped into multi-site compounds, each will exhibit various luminescence performances. K_7_ZnSc_2_B_15_O_30_:Mn^2+^ shows two emissions peaked at 584 nm and 675 nm due to the divalent Mn doping into the Zn^2+^ and Sc^3+^ sites in the host [82]. MgGa_2_O_4_:Mn^4+^ exhibits asymmetric emission due to the tetravalent Mn doping into the Mg^2+^ and Ga^3+^ sites, whose emission bands can be divided into two sub-bands centered at 674 nm and 710 nm, respectively [83]. Incorporating Mg^2+^/Ge^4+^ regulates the Mg/Ga anti-site in the lattice, resulting in the improvement of emission intensity by 1.6 times for Mg_2.7_Ga_2.6_Ge_0.7_O_8_:Mn^4+^ with a FWHM of 69 nm. Furthermore, the luminescent regulation of multi-valence Mn ions can be realized by tuning the local structure to the multi-site preference. In the double perovskite-structure Ca_2_MgWO_6_ lattice, the Mn ions prefer to dominate the Mg^2+^ site and stabilize at a divalent state due to the appropriateness in ionic radii and valence state [84]. To obtain the Mn^4+^-related far-red emission, the Na^+^-La^3+^ pair are introduced to replace the Ca^2+^-Ca^2+^ pair; this leads to the contraction of [MgO_6_] and the enlargement of the [WO_6_] octahedron, as shown in Figure 8a. The structural distortion induces the migration of Mn ions from Mg^2+^ to W^6+^ sites and results in the effective Mn^4+^ emission, as illustrated in Figure 8b. The internal quantum efficiency of these samples reaches to 94%, showing excellent luminescent performance, as shown in Figure 8c.

#### 2.2.6. Bi^3+^

Recently, a series of Bi^3+^-activated phosphors with strong near-ultraviolet absorption and efficient emission have been developed for high-quality pc-LEDs. Based on the multi-site luminescence strategy, a broadband cyan-emitting K_2_Ca_2_Si_2_O_7_:Bi^3+^ phosphor is designed and synthesized [85]. The K_2_Ca_2_Si_2_O_7_ lattice provides three possible crystallographic sites for Bi^3+^ ions. When the Bi^3+^-doping concentration is small, the activator ions prefer to dominate the two types of Ca^2+^ sites. When the Bi^3+^-doping concentration increases, the emission spectra can be deconvoluted into three sub-bands corresponding to the two types of Ca^2+^ sites and the K(6) site. According to the characteristic transition of Bi^3+^, some novel Bi^3+^-activated phosphors with multi-site luminescence have been developed to fill the cyan gap for full-spectrum wLEDs [86].

**Figure 9:**
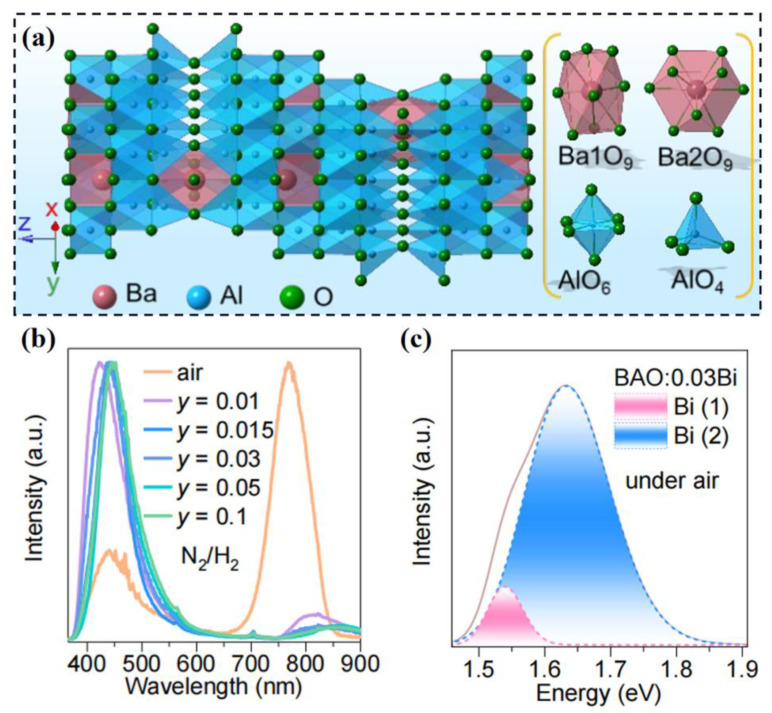
(**a**) Schematic crystal structure diagram of BaAl_12_O_19_ matrix. (**b**) PL spectra of BaAl_12_O_19_:Bi^3+^-air and BaAl_12_O_19_:yBi^3+^-N_2_-H_2_ (0 ≤ y ≤ 0.10). (**c**) Gaussian fitting PL spectra of BaAl_12_O_19_:Bi^3+^-air (λ_ex_ = 330 nm). Reprinted with permission from ref. [87]. Copyright 2020 American Chemical Society.

Furthermore, a novel near-infrared Bi^3+^-activated phosphor based on selective site occupation has been developed [87]. The BaAl_12_O_19_ matrix with a hexagonal structure provides two kinds of Ba sites and five kinds of Al sites, which form Ba1O_9_, Ba2O_9_, AlO_6_ and AlO_4_ polyhedrons, as illustrated in Figure 9a. The Bi^3+^-activated BaAl_12_O_19_ phosphor shows blue or NIR emission under different synthetic atmosphere conditions, as shown in Figure 9b. The BaAl_12_O_19_:Bi^3+^-N_2_-H_2_ sample exhibits blue emission peaked at 440 nm. The reduced synthetic atmosphere stabilizes oxygen vacancy, which leads to the preferential occupation of Bi^3+^ at two types of Ba^2+^ sites. However, the BaAl_12_O_19_:Bi^3+^-air sample exhibits unique NIR emission, whose PL spectrum can be divided into two sub-bands due to the Bi^3+^ dominated at AlO_6_ and AlO_4_ polyhedrons, as shown in Figure 9c. The air atmosphere condition results in the appearance of rare oxygen vacancy, so that the majority occupation of Bi^3+^ at Al^3+^ keeps the charge balance. Hence, the synthetic atmosphere induced multi-site luminescence modification will be a promising design strategy for novel phosphors.

## 3. Identification of Multisites for the Activator Ions

The local crystal environment where the activator ions are doped directly decides the multi-site luminescence performance. Clarifying the multisites for the activator ions is the key to investigating the luminescent mechanism, guide the design of novel materials and inspire the modification of their luminescent properties. Generally, the activator ions prefer to occupy the cations with similar ionic radii and valence state in the lattice. However, as with the multi-site host lattice, the various crystallographic sites where activator ions are doped can be complex and flexible. To intensively study the identification of the preferential occupation, several measurements and calculation methods have been reported, including some optical analysis methods (low-temperature PL, transient fluorescence spectroscopy, lattice probe, etc.), structural analysis methods (refinement of X-ray diffraction ‘XRD’, electron paramagnetic resonance ‘EPR’, X-ray photoelectron spectroscopy ‘XPS’, etc) and theoretical calculations (First Principle calculation, occurrence probability, Judd-Ofelt theory, etc.). The following section will introduce some research about the identification of multisites for the activator ions.

### 3.1. Optical Analysis

**Figure 10:**
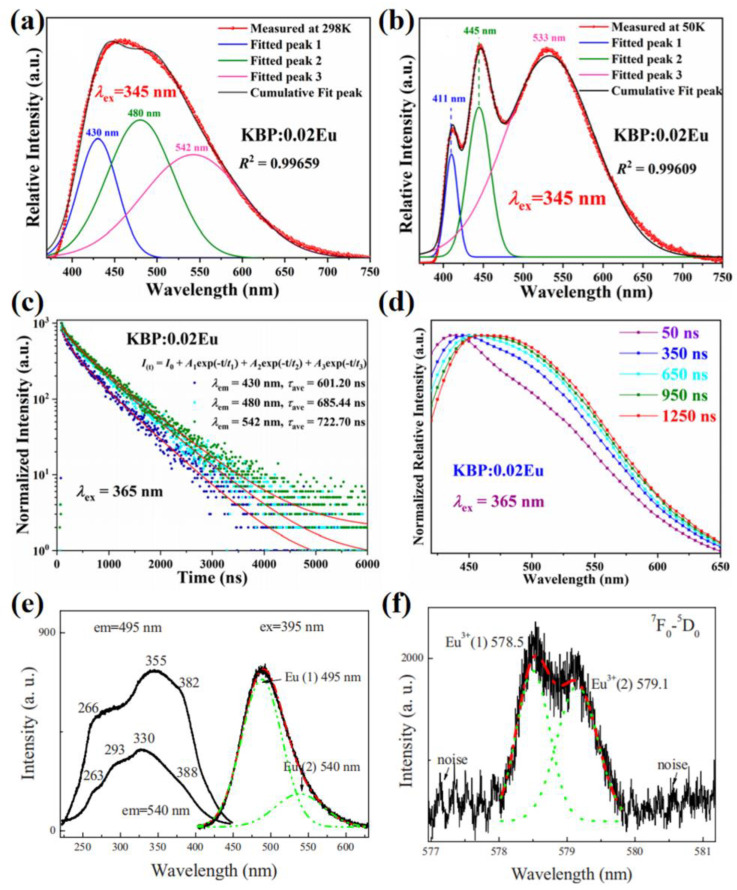
Fitted Gaussian components of the (**a**) emission spectrum at 298 K, (**b**) emission spectrum at 50 K, (**c**) comparison of the different emission decay curves with identical excitation (430, 480 and 542 nm), (**d**) normalized spectral slices of TRPL at 50, 350, 650, 950 and 1250 ns of the KBa_2_(PO_3_)_5_:0.02Eu. Reprinted with permission from ref. [39]. Copyright 2020 American Chemical Society. (**e**) The emission and excitation spectra of KCaPO_4_:Eu^2+^. (**f**) The excitation spectra in the region of ^7^F_0_→^5^D_0_ transition in KCaPO_4_:Eu^2+^. Reprinted with permission from ref. [88]. Copyright 2010 Electrochemical Society (Pennington, NJ, USA).

In general, the emission spectra of multi-site phosphors show asymmetric bands that can be divided into several Gaussian sub-peaks. Multi-site occupations in the host lattice directly determine the transitions of activator ions; in addition, the optical performance can reflect the local structure. The low-temperature PL spectra removes the effects of thermal vibration and represents the intrinsic emission of activators, which are usually measured to analyze the multi-site luminescence. Figure 10a,b show the photoluminescence spectra of KBa_2_(PO_3_)_5_:0.02Eu measured at 298 K and 50 K, respectively [39]. The three Gaussian components contributed to the Eu activator ions dominated at the K1, Ba1 and Ba2 sites. The decay curve of the sample is better fitted with the triple-exponential model, that shows three crystallographic sites of Eu, as shown in Figure 10c. To further investigate the activator occupation, the time-resolved photoluminescence (TRPL) spectrum is measured. It shows a slower decay in the lower-energy region than in the higher-energy region. The PL spectra at different temperature and transient fluorescence spectroscopy can be used to identify the multiple luminescence centers [37]. The fitted lifetime monitored at different wavelengths obtained by the decay curve measurements can represent the preferential occupation of the activator ions among the several luminescence centers. As for the (Ba,Sr)_9_Lu_2_Si_6_O_24_:Eu^2+^ phosphor mentioned in the former section, the lifetimes under different excited wavelengths are almost unchanged for the Sr-free sample, whereas the lifetimes increase with the increase of excited wavelengths for the Sr-substituted samples [26]. The different tendency of the lifetimes exhibits the variety of preferential occupation of Eu^2+^ induced by the Sr-substitution in the host lattice.

Due to the non-degenerate energy levels, the electric dipole ^5^D_0_→^7^F_0_ transition will not split with the crystal field interaction [89]. Hence, the Eu^3+^ ion can be applied as the lattice probe to identify the multisites of Eu or other ions in the lattice. Figure 10e shows the two sub-bands of the emission spectrum for the KCaPO_4_:Eu^2+^ [88]. The Eu-activated phosphor is synthesized as the lattice probe material, whose excitation spectrum consists of two transition lines corresponding to the two crystallographic sites of Eu^3+^, as illustrated in Figure 10f. Although the local structures around the divalent and trivalent Eu ions cannot be the same due to the different charge compensation, the above evidences could prove that the Eu^2+^ activator ions dominate the two kinds of Ca^2+^ sites in the KCaPO_4_ lattice.

### 3.2. Structural Analysis

**Figure 11:**
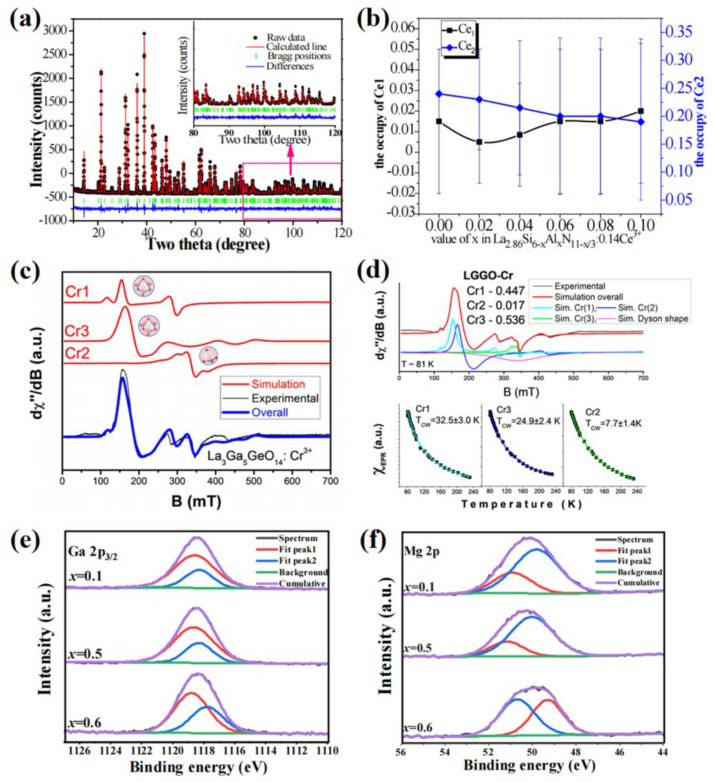
(**a**) Rietveld refinement of the powder data of La_2.86_Si_5.9_Al_0.1_N_10.97_:0.14Ce^3+^. (**b**) Ce occupancy of La_2.86_Si_6−x_Al_x_N_11−x/3_:0.14Ce^3+^ samples from the refinements. Reprinted with permission from ref. [92]. Copyright 2017 Royal Society of Chemistry. (**c**) EPR spectrum registered for La_3_Ga_5_GeO_14_ compound doped with Cr^3+^. (**d**) Deconvoluted EPR spectrum and its temperature dependence in La_3_Ga_5_GeO_14_:Cr^3+^. Reprinted with permission from ref. [94]. Copyright 2020 American Chemical Society. High-resolution XPS spectra of Mg_2+x_Ga_4−2x_Ge_x_O_8_:Mn^4+^ samples: (**e**) Ga 2p_3/2_ and (**f**) Mg 2p. Reprinted with permission from ref. [83]. Copyright 2021 Elsevier B.V.

Crystal diffraction is a classic measurement used to investigate structural analysis, such as X-ray diffraction, neutron diffraction, etc. [90]. The refinements of crystal diffraction are an effective way to obtain detailed structural information. To investigate the doping ions in the lattice, constructing several possible doping models and comparing their deviation values from the refinement results can reveal the preferential occupation of the doping ions [91]. The lower the deviation value, the more preferred the doping models. These fitting methods have been applied to the identification of multisites for the activator ions by many researchers [26,63]. The refinement results can provide the accurate occupancy of the activator at different crystallographic sites [92,93]. Figure 11a exhibits the Rietveld refinement result of La_2.86_Si_5.9_Al_0.1_N_10.97_:0.14Ce^3+^. The Ce^3+^ ions are dominated at two kinds of La sites; their occupancy at different sites can be calculated via the Rietveld refinements, as shown in Figure 11b. The results show that the Ce^3+^ ions prefer to enter the La2 site with the Al^3+^ doping.

The general testing technologies for structural analysis can be used to confirm the occupation of multi-site luminescent materials, including EPR, XPS, etc. Figure 11c,d illustrates the EPR results, which are measured to clarify the local environment of Cr^3+^ in the La_3_Ga_5_GeO_14_ lattice [94]. The EPR spectrum is deconvoluted into three different spectra due to different luminescent centers (Cr1, Cr2 and Cr3). The EPR signal of Cr2 at a middle magnetic induction consists of broad asymmetric lines with a *g* value of nearly 1.99, whereas the signals of Cr1 and Cr3 at a low magnetic induction consist of broad asymmetric lines with *g* values of around 4.38 and 4.12, respectively. The divided individual Cr centers are calculated as Cr1 of 44.7%, Cr2 of 1.7% and Cr3 of 53.6%. The Dyson line in the EPR spectra clarifies the shape of the temperature dependent integral intensity for the phosphor. The multi-site occupation of activator ions can be confirmed using XPS measurement [71]. The XPS spectra of Mg_2+x_Ga_4−2x_Ge_x_O_8_:Mn^4+^ samples reveal asymmetric bands, as the larger binding energy peak is attributed to the cations dominated at octahedron and the smaller binding energy peak is contributed to the cations dominated at tetrahedron. This is illustrated in Figure 11e,f.

### 3.3. Theoretical Calculation

**Figure 12:**
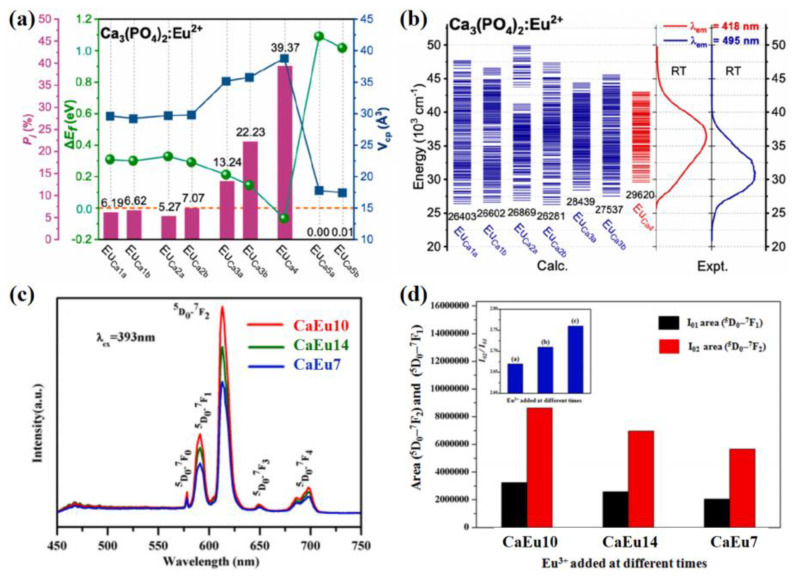
(**a**) Calculated defect formation energies (Δ*E_f_*), occurrence probabilities (*P_j_*) and volumes of relaxed coordination polyhedra (*V_cp_*) for Eu^2+^ substitutions at various Ca sites in Ca_3_(PO_4_)_2_. (**b**) Schematic diagram for the calculated 4f^6^5d^1^ energy levels of Eu^2+^ in Ca_3_(PO_4_)_2_. Reprinted with permission from ref. [95]. Copyright 2022 American Chemical Society. (**c**) The PL emission spectra. (**d**) area (^5^D_0_-^7^F_2_), area (^5^D_0_-^7^F_1_) and I_02_/I_01_ (inset) for PL emission spectra of the series of CaCO_3_:Eu^3+^ samples. Reprinted with permission from ref. [96]. Copyright 2021 Elsevier B.V.

Constructing several possible doping models and comparing their formation energy using first principle DFT calculations can investigate the preferential occupation of the doping ions [91]. The lower formation energy means greater stability for the corresponding doping models, that can reveal the occupation of activator ions in the multi-site phosphors. Figure 12a depicts the formation energies (Δ*E_f_*) for Eu^2+^-substitution at the Ca sites for various structural models [95]. The calculations show that Eu ions prefer to occupy the Ca4 site, followed by the Ca3 and the Ca1/Ca2 sites in turn; however, they do not prefer to occupy the Ca5 site in the Ca_3_(PO_4_)_2_ lattice. Corresponding to the calculated energy levels of Eu^2+^ shown in Figure 12b, the luminescence of Ca_3_(PO_4_)_2_:Eu^2+^ can be deconstructed as the 418 nm emission due to Eu^2+^ at the Ca4 site. The broadband emission peaked at 630 nm attributed to Eu^2+^ at the Ca1-Ca3 site, and the shorter and longer-wavelength components are caused by Eu^2+^ at the Ca3 and the Ca1/Ca2 sites, respectively. The occurrence probability (*P_j_*) can be evaluated to quantify the relative preference of the activator ions doping, which can be measured by the formula $P_{j}=\frac{1}{z_{tot}}\Omega_{i}exp\left(-\frac{E_{i}}{kT}\right)$ [34]. In the formula, $z_{tot}$ represents the partition function, $\Omega_{i}$ represents the multiplicity, *k* is the Boltzmann constant, $E_{i}$ represents the relative total energy of the unit cell and *T* is the synthesis temperature. The *P_j_* values for Eu^2+^ located at the five types of Ca sites are 12.81%, 12.34%, 35.47%, 39.37% and 0.01%, respectively. The calculations of formation energy and occurrence probability both prove the occupying information for the multi-site phosphors. Especially when Eu^3+^ is the activator, the Judd–Ofelt theory can be used to investigate symmetry, coordination environment and luminescence behavior [96]. Figure 12c exhibits the PL emission spectra of a series of CaCO_3_:Eu^3+^ samples. Figure 12d illustrates the areas of ^5^D_0_-^7^F_2_ and ^5^D_0_-^7^F_1_ for PL emission spectra. As for the luminescence behavior of Eu^3+^, the total relative integrated intensity (I=∑J=0−6FJ7) is proportional to the total radiative transition rate for ^5^D*_0_*-^7^F*_J_* transition. The relevant computational formulas are complex, and are not expressed one by one. As for the Judd–Ofelt parameters of the series of CaCO_3_:Eu^3+^ samples, *Ω*_2_ is related to the local symmetry around Eu^3+^ and the covalency between Eu^3+^ and O^2−^, and *Ω*_4_ is related to the bulk properties as a long-range effect but not to the local structure. Hence, the calculated results prove that the samples treated at different conditions exhibit different occupation of the Eu^3+^ ion on the surface or in the bulk of the optical materials.

## 4. Regulation of Multi-Site Luminescence

Multi-site luminescence is determined by the crystal structure of the phosphors. The regulation of multi-site luminescence can be achieved via cation or anion substitution, solid solution substitution and energy transfer, among other methods. Through the multi-site regulation strategy, the emission peaks, intensities, shapes and FWHM can be modified, the luminescence performance can be improved and novel luminescent materials and advanced application can be realized.

### 4.1. Modification of Emission Spectrum

**Figure 13:**
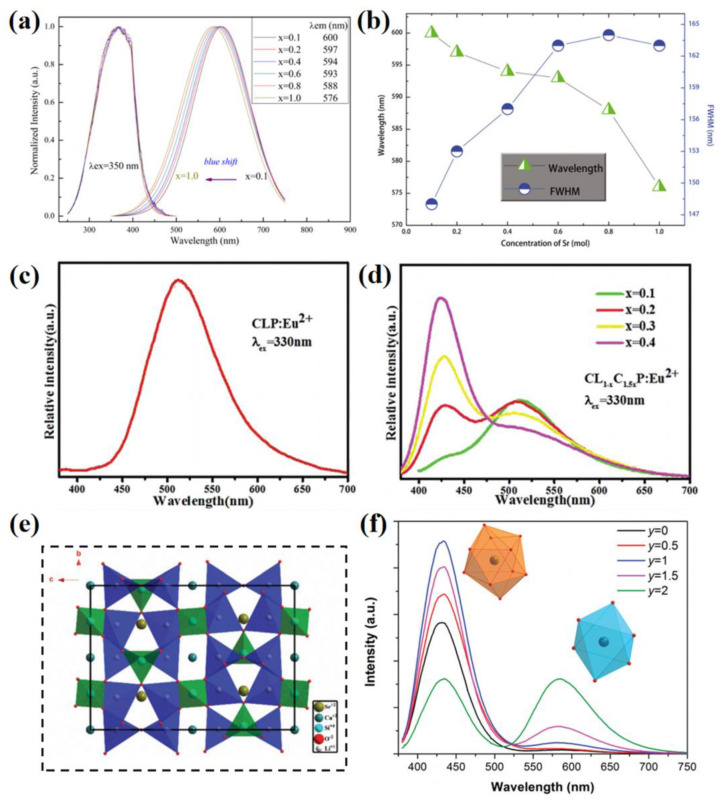
(**a**) Intensities normalized PLE and PL spectra. (**b**) Peak wavelength and the FWHM of samples with nominal formula Ba_0.98(2−x)_Sr_0.98−x_Mg(PO_4_)_2_:0.04Eu^2+^ (x = 0.1, 0.2, 0.4, 0.6, 0.8, 1.0). Reprinted with permission from ref. [38]. Copyright 2015 American Ceramic Society. Emission spectra of (**c**) Ca_9_La(PO_4_)_7_:Eu^2+^ and (**d**) Ca_9_La_1−x_Ca1.5_x_(PO_4_)_7_:Eu^2+^. Reprinted with permission from ref. [89]. Copyright 2019 Royal Society of Chemistry. (**e**) The crystal structure diagram of Li_4_SrCa(SiO_4_)_2_. (**f**) Emission spectra of Li_4_SrCaSi_2_O_8−2y_N_4y−3_:0.05Eu^2+^ phosphors (λ_ex_ = 363 nm). Reprinted with permission from ref. [98]. Copyright 2022 Royal Society of Chemistry.

Generally, multi-site luminescent materials exhibit broadband emission, as shown in Section 2. The activator ions dominated at different crystallographic sites show different luminescence processes that combine to obtain the emission band with a large FHWM. By the modification of the multi-site structure, the emission band is able to be broadened further, allowing it to be used for high-quality full-spectrum lighting, near-infrared lighting, single-component white emitting phosphor, etc. [26,59,97]. For example, Ba_2_Mg(PO_4_)_2_:Eu^2+^ is a typical phosphate phosphor material used for full-spectrum wLEDs. It possesses two kinds of crystallographic sites of Ba^2+^ and exhibits a broad yellow emission [38]. The Sr-substitution is employed to construct multi-site occupation for the Eu^2+^, as the activator ions randomly occupy two Sr^2+^ and two Ba^2+^ crystallographic sites. Due to the Sr-substitution in the lattice, the multi-site luminescence is tuned, as shown in Figure 13a. As for the detailed luminescent information in Figure 13b, the unusual blue shift of the emission peak shift from 600 to 576 nm is due to the changes of cell volume and bond distances; the FWHM broadens from 148 nm to 163 nm due to the multi-site occupancy of the activator ions. The wider emission characteristic means the Sr-substituted samples can be widely applied.

**Figure 14:**
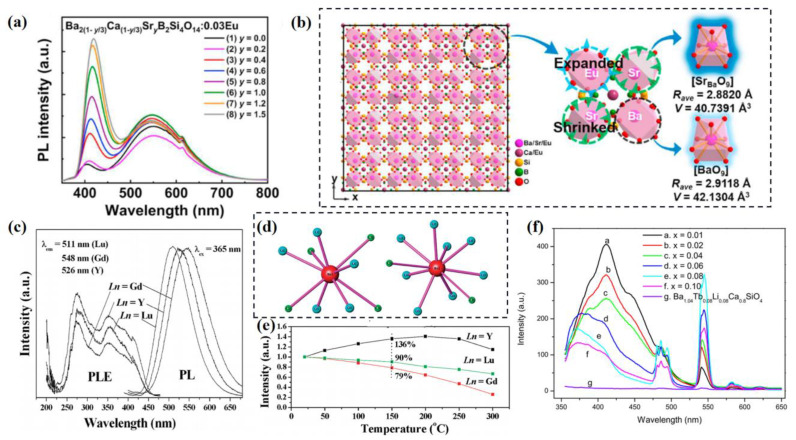
(**a**) PL spectra of Ba_2(1−y/3)_Ca_(1−y/3)_Sr_y_B_2_Si_4_O_14_:Eu (0 ≤ y ≤ 1.5) phosphors (λ_ex_ = 318 nm). (**b**) Schematic diagram of structural evolution after cationic Sr substitution. Reprinted with permission from ref. [34]. Copyright 2022 Elsevier Inc. (**c**) PLE and PL spectra under 365 nm UV light and (**e**) temperature-dependence of the integrated emission intensities of Ba_2_Ln(BO_3_)_2_Cl:Eu^2+^ (Ln = Y, Gd and Lu) phosphors. (**d**) Coordination spheres of the two different Ba^2+^ positions in Ba_2_Ln(BO_3_)_2_Cl compounds. Reprinted with permission from ref. [99]. Copyright 2011 American Chemical Society. (**f**) Tb^3+^ concentration dependence of emission spectra for Ce^3+^, Tb^3+^ co-doped Ba_1.16−2x_Ce_0.02_Tb_x_Li_0.02+x_Ca_0.8_SiO_4_ samples and Tb^3+^-doped Ba_1.04_Tb_0.08_Li_0.08_Ca_0.8_SiO_4_ sample (λ_ex_ = 345 nm). Reprinted with permission from ref. [100]. Copyright 2018 Elsevier B.V.

Generally, the cation substitution strategy can directly regulate the local structure where the activator ions are doped. This has been the classic method for the modification of luminescence performance. As for the multi-site luminescent materials, cation substitution results in more varied changes and can even induce the activator ions to occupy some seemingly impossible crystallographic sites. For instance, the Ca_9_La(PO_4_)_7_ lattice possesses three different Ca sites and one La site (Ca1, Ca2, Ca3 and La, respectively [89]). Because of the similar lattice environment and valence state, the Eu^2+^ activator ions prefer to dominate the three Ca sites to show multi-site luminescence as an asymmetrical broadband emission, shown in Figure 13c. Based on the calculations of the radius percentage difference, the results prove that the Ca^2+^ can be successfully introduced into the only La^3+^ site in the lattice. The Ca^2+^-cation-substitution leads to the Eu^2+^ activator ions being doped at the La^3+^ site; this exhibits significant changes to the shapes of the emission band, as illustrated in Figure 13d. Similarly, other divalent alkaline earth metal ions are introduced into the lattice to replace the lanthanide; these create potential crystallographic sites for the Eu^2+^ activator ions and achieve effective spectral regulation in the multi-site phosphors. Moreover, an anion substitution strategy can also affect the local structure of the multisites for the activator ions. The Li_4_SrCa(SiO_4_)_2_ lattice provides one kind of Ca site and one kind of Sr site; these can be dominated by the Eu^2+^ activator ions, as shown in Figure 14e [98]. A multi-site luminescence with the combination of a blue emission and an orange emission is observed. With the substitution of N^3−^ for O^2−^, the Eu-O polyhedron is distorted and the preferential occupation of Eu^2+^ is changed. The result is that the blue emission gradually decreases as the orange emission gradually enhances, as shown in Figure 14f. Hence, the multi-site regulation strategy can modify the emission spectrum to achieve various luminescent performances.

### 4.2. Enhancement of Luminescence Performance

The regulation strategy can also be used to improve the luminescence performance of multi-site phosphors, including emission intensity, quantum efficiency, thermal stability, etc. The unique borosilicate Ba_2_CaB_2_Si_4_O_14_:Eu^2+^, with a tetragonal crystal structure, possesses two different crystallographic sites for the activator ions, whose emission spectrum consists of two emission bands peaked at 408 nm and 548 nm due to the Eu^2+^ dominated at the Ba^2+^ and Ca^2+^ sites, respectively [34]. With the substitution of Sr into the lattice, the emission intensity at 408 nm increases significantly, while that at 548 nm changes only slightly, as shown in Figure 14a. According to the structural analysis, the Sr-substitution primarily influences the luminescence of activator ions at the Ba^2+^ site; this shows that the local structural contraction around Sr^2+^ results in the expansion and stretch of the [EuO_9_] polyhedron, as illustrated in Figure 14b. The structural evolution of chemical pressure release leads to the enhancement of local crystal field environments, and eventually causes the improvement of the emission intensity of multi-site luminescence. The quantum yield is improved from 17.9% to 36.8%, and this series of phosphors are promising candidates for single-component white emission.

Thermal stability is an essential parameter to consider when evaluating the application performance of phosphors. It is determined by the structure rigidity, band gap, crystal defects and other local structures of the luminescent materials [101]. The evolution of multi-site structure can achieve the enhancement of thermal stability. As shown in Figure 3d, an appropriate Sr-substitution into the Ba_9_Lu_2_Si_6_O_24_:Eu^2+^ lattice can improve the thermal stability because the Eu^2+^ ions prefer to dominate the Ba2 site with higher rigidity, gradually increasing the Sr-substituted concentration from 0 to 0.3 [26]. However, as the Sr-substituted concentration increases further, the Eu^2+^ ions are induced to partially occupy the Ba3 site with lower rigidity, which results in lower thermal stability. As for the multi-site phosphors, different crystallographic sites for the activator ions exhibit different local structure characteristics, meaning the regulation of local structure affects thermal performance. As for Eu^2+^-activated Ba_2_Ln(BO_3_)_2_Cl, their emission spectra are centered at 526, 548 and 511 nm, as shown in Figure 14c, with the FWHM of 97, 108 and 91 nm for the Ln = Y, Gd and Lu, respectively [99]. Their broadband emissions are attributed to the multisites for the activator ions, as with the Ba1 and Ba2 sites, as illustrated in Figure 14d. As for the Ln = Gd and Lu samples, the emission intensity decreases gradually as the temperature increases. However, the Ln = Y sample exhibits an abnormal thermal phenomenon; the emission intensity increases and then declines slightly when temperature is more than 200 °C, as shown in Figure 14e. The special thermal phenomenon is attributed to the different luminescence efficiency of the multi-site emission centers, which is further proved by the theoretical calculation of the activation energy of thermal quenching. Furthermore, constructing energy transfer is an effective way to enhance the multi-site luminescence performance. As shown in Figure 14f, the emission intensity of Tb^3+^ in Ba_1.2_Ca_0.8_SiO_4_ can be improved by 30 times due to the co-doping of Ce^3+^ and Tb^3+^ [100]. The multi-site luminescence of Ce^3+^ in the lattice induces the energy transfer from Ce^3+^ to Tb^3+^, which significantly enhances the luminescence performance of the co-doped phosphors.

### 4.3. Optimization of Temperature-Dependent Sensitivity

Besides high-quality lighting for wLEDs, multi-site luminescence has also been applied in non-contact optical thermometric technology for temperature measurements in thermally harsh, corrosive or intracellular environments [102]. Initially, the temperature sensing property is revealed by the double emission intensity ratio between the thermally coupled energy levels (TCELs) of lanthanide ions, such as Er^3+^, Ho^3+^, Tm^3+^, Sm^3+^, Yb^3+^, Dy^3+^, Gd^3+^, Nd^3+^, etc. [36]. However, the difference of their TCELs is too narrow to obtain high sensitivity and strong recognition ability. To further improve the optical thermometric property, the multi-site luminescence of Eu^2+^, Ce^3+^, Cr^3+^, Mn^2+^ or other kinds of activators that are susceptible to the external crystal field gradually attracted more attention [48,82]. Hence, many relevant studies concerning the application of multi-site phosphors for optical thermometric properties, that have been mentioned many times before, have been reported recently.

**Figure 15:**
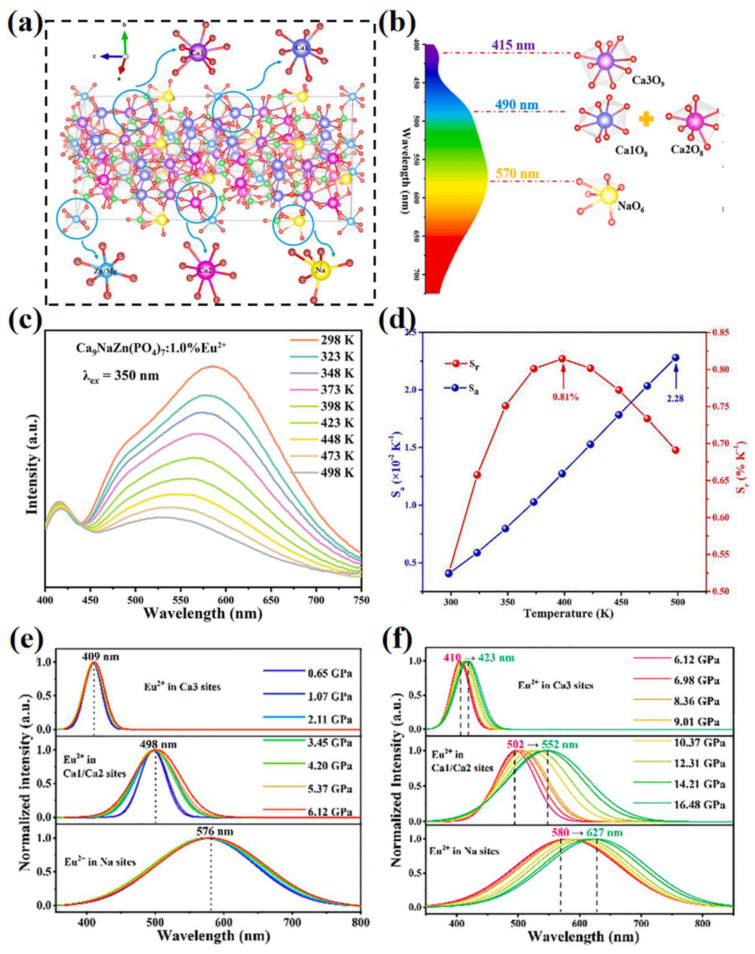
(**a**) Crystal structure of the Ca_9_NaZn(PO_4_)_7_. (**b**) PL spectrum corresponding to different cationic sites of Ca_9_NaZn(PO_4_)_7_:1.0%Eu^2+^ phosphor excited by 350 nm. (**c**) Temperature-dependent PL spectra, (**d**) the calculated plots of absolute sensitivity (S_a_) and relative sensitivity (S_r_) versus temperature, (**e**,**f**) normalized Gaussian fitting PL spectra for Eu^2+^ ions at different cation sites under different pressure of Ca_9_NaZn(PO_4_)_7_:1.0%Eu^2+^ phosphor. Reprinted with permission from ref. [105]. Copyright 2021 Elsevier B.V.

According to the requirements of the non-contact optical thermometric technology, multi-site luminescence should be characterized by fast response, high precision and high spatial-temporal resolution [102,103]. Based on the temperature measurement related to the fluorescence intensity ratios (FIR), temperature-dependent absolute sensitivity (S_a_) and relative sensitivity (S_r_) can represent the optical thermometric property of the phosphors [35,98]. The measurement methods and calculation formula will not be repeated here, as they can easily be found in several papers [104]. The temperature-dependent sensitivity can exhibit the applicable temperature range and potential. For example, Ca_9_NaZn(PO_4_)_7_ is an multi-site structure for luminescence (crystal structure model is shown in Figure 15a) that provides a variety of possibilities for the activator ions, including three types of Ca sites (Ca1, Ca2 and Ca3), an Na site, etc. [105]. As shown in Figure 15b, the Eu^2+^-doped Ca_9_NaZn(PO_4_)_7_ phosphor exhibits an asymmetric broad emission band with peaks at around 415, 490 and 570 nm due to the 5d-4f transition of Eu^2+^ at Ca3, Ca1/Ca2 and Na sites, respectively. The Mg^2+^-substitution in the Ca_9_NaZn(PO_4_)_7_:Eu^2+^ phosphor adjusts the emission from yellow to warm white via an increased red-emitting component; this achieves good optical properties for wLEDs. The three sub-emissions of the multi-site luminescence shows different temperature-dependent performance, as shown in Figure 15c. As the temperature increases, the emission intensity of Eu^2+^ in Na site decreases significantly, and the Eu^2+^ at Ca3 site changes slightly. This might be attributed to the local crystal structure of each crystallographic site. According to the FIR calculation, the maximum S_a_ is 0.0228 K^−1^ at 498 K and the maximum S_r_ is 0.81 K^−1^ at 398 K (as shown in Figure 15d). This confirms the promising application of the Ca_9_NaZn(PO_4_)_7_:Eu^2+^ phosphor for optical thermometry. Interestingly, the Ca_9_NaZn(PO_4_)_7_:Eu^2+^ phosphor is sensitive not only temperature, but also pressure, as shown in Figure 15e,f. With an increase of pressure from 0.65 to 6.12 GPa, the FWHM of emission bands for Eu^2+^ in Ca1/Ca2 sites are enlarged gradually. With an increase of pressure from 6.12 to 16.48 GPa, the emission bands and their intensities for Eu^2+^ in three luminescence centers are regulated significantly. In addition, the peaks shift from 410 to 423 nm, 502 to 552 nm and 580 to 627 nm for Eu^2+^ in the Ca3, Ca1/Ca2 and Na sites, respectively. The reduced symmetry, an increase in generated defects and increased phonon energy caused by pressure increasing lead to the broadening of FWHMs, as the larger nephelauxetic effect and stronger crystal field splitting due to structural shrinkage by pressure increase results in the red-shift of emission peaks. The Ca_9_NaZn(PO_4_)_7_:Eu^2+^ phosphor shows a better pressure sensitivity (5.21 nm/GPa) and exhibits very close CIE chromaticity parameters under the same pressure during the compression and decompression processes. This demonstrates its potential application for optical pressure sensors.

## 5. Summary and Outlook

Our review describes the construction, identification and regulation of the multi-site luminescence based on research and investigations conducted over the past 20 years. First, several types of host materials with various crystallographic sites for activator ions to construct multi-site phosphor are introduced in detail, and multi-site luminescence characteristics of different activator ions are summarized, including Eu^2+^, Ce^3+^, Cr^3+^, Eu^3+^, Mn^2+^, Mn^4+^, Bi^3+^, etc. Second, identification of the preferential occupation for the activator ions is difficult but essential to reveal the structural characteristics and modify the optical performance for the multi-site phosphors. Using optical analysis, structural analysis and theoretical calculations, several measurements and methods are evaluated and elucidated to clarify the multi-site luminescence. Third, based on the clear understanding of the structure and optical properties, the regulation strategies of multi-site luminescence and their representative work are introduced and discussed in terms of modification of emission spectrum, enhancement of luminescence performance, applications on advanced fields, etc. According to the review of various experimental, theoretical and applied results, we conclude that there are still several important and necessary challenges for multi-site luminescence that must be investigated and developed in future, including but not limited to the following:

(1) Design of a novel multi-site phosphor with excellent luminescent performance is still an ongoing mission. Achieving delicate control of emission color during the design process is essential for the material science. Recently, several approaches have been carried out to discover new materials based on known or unknown crystal structures that can be used to find multi-site hosts, such as solid-state combinatorial chemistry, single crystal growth, single-particle-diagnosis, etc. Furthermore, materials genome engineering and big data computing can be introduced to design a novel multi-site phosphor. Based on a high-quality material database and using scientific and accurate criteria, high throughput screening and experimental verification can effectively achieve the development of new luminescent materials. In the above approach, the mineral structure model, phase diagram, thermodynamics, energy band theory and other basic theories can guide the screening and optimization of new materials.
(2) Optimization of luminescence properties of multi-site phosphors is still an important challenge. An excellent phosphor material should have strong absorption, strong emission, high luminous efficiency, high stability, etc. Generally, delicate compositional tailoring is a valid strategy to modify the surrounding crystal environment of activator ions to enhance luminescence performance, such as cation substitution, anion substitution, solid solution substitution, constructing energy transfer, etc. However, the origin of multi-site luminescence is complicated; as such, accurate directional regulation is difficult. The first principles calculation equipped with high-throughput computation and machine learning will be helpful to effectively analyze the relationship of structure and properties, which could be a promising strategy for optimization of multi-site luminescent materials.
(3) Achieving wider and deeper advanced applications of multi-site phosphors is an eternal goal. Multi-site luminescent materials have been used in high-quality full-spectrum lighting due to their broad and tunable emission. Many multi-site NIR phosphors have been developed for use in security monitoring, food composition analysis, bio-sensing, etc. Recently, the applications in non-contact temperature testing have attracted significant attention and have been summarized specifically. The emission intensity and material stability of these applied phosphors must be improved further. Multi-site phosphors with better performance will obtain wider and deeper advanced applications, and could be customized according to the specific multi-site luminescence characteristics.

With the higher requirements of applications, the study and research of multi-site luminescence will never stop. We hope our review will provide some inspiration for the design and modification of the multi-site phosphors, even in the wider field of luminescent materials or other materials science.

## Figures and Tables

**Table 2:** Summary of structural and luminescent information for some Ce^3+^-doped multi-site phosphors.

Host	Multisites (The Emission Peaks)	FWHM	Reference
Ca_3_Sc_2_Ge_3_O_12_	Ca (490 nm), vibration-coupling structure (530 nm)	-	[23]
Ba_2_Y_3_[SiO_4_]_3_	Ba/Y1 (405, 440 nm), Ba/Y2 (505, 565 nm)	7130 cm^−1^	[49]
Ba_2_CaB_2_Si_4_O_14_	Ba (399, 431 nm), Ca (473, 523 nm)	142 nm	[50]
Sr_3_Si_13_Al_3_O_2_N_21_	three kinds of Sr (453 nm)	-	[51]
NaCaBO_3_	Ca1 (387, 419 nm), Ca2 (432, 469 nm)	-	[52]
Ca_10_Li(PO_4_)_7_	Ca1/Ca2/Ca3 (330, 355 nm), Ca5 (430, 460 nm)	-	[53]

**Table 3:** Summary of structural and luminescent information for some Cr^3+^-doped multi-site phosphors.

Host	Multisites (The Emission Peaks)	FWHM	Reference
Ca_3_Ga_2_Ge_3_O_12_	Ca (749 nm), Ga (803 nm), Ge (907 nm)	-	[22]
Ca_3−x_Lu_x_Ga_2+x_Ge_3−x_O_12_ (x = 0–1)	Ga, Ca (766–803 nm)	129–267 nm	[65]
Mg_3_Ga_2_GeO_8_	Ga1(760 nm), Ga2 (842 nm), Ga3 (918 nm), Ga4 (960 nm)	-	[66]
Mg_7_Ga_2_GeO_12_	[Ga1/Mg1] (700 nm), [Ga3/Mg3, Ga4/Mg4] (723 nm), [Ga2/Mg2] (778 nm), [Ga5/Ge1] (867 nm)	101–226 nm	[64]
La_3_Ga_5_GeO_14_	Ga1, Ga3 (980 nm)	330 nm	[63]
Ga_4_GeO_8_	Ga1, Ga2, Ga3 (850 nm)	215 nm	[67]
LaMgGa_11_O_19_	Ga1, Ga4, Ga5	133 nm	[68]
SrGa_12_O_19_	Ga1, Ga4, Ga5	83 nm	[68]
La_2_MgZrO_6_	Mg, Zr (825 nm)	210 nm	[69]
Li_2_Mg_3_TiO_6_	Mg, Ti (720–920 nm)	258 nm	[70]
Li_3_Mg_2_NbO_6_	Mg1 (714 nm), Mg2 (744 nm), Mg3 (809 nm)	120 nm	[71]
LiMgGaF_6_	Ga (791 nm), Mg (875 nm)	189.9 nm	[72]
Li_3_Sc_2_(PO_4_)_3_	Sc1 (934 nm), Sc2 (978 nm)	248 nm	[73]
ABO_4_ (A: Ga, Sc, In; B: Ta, Nb)	A, B (825–1025 nm)	125–231 nm	[74]
AlNbO_4_	Al1, Al2	244–322 nm	[75]

## Data Availability

No new data were created.
